# The Advisory Committee on Immunization Practices’ Interim Recommendation for Use of Janssen COVID-19 Vaccine — United States, February 2021

**DOI:** 10.15585/mmwr.mm7009e4

**Published:** 2021-03-05

**Authors:** Sara E. Oliver, Julia W. Gargano, Heather Scobie, Megan Wallace, Stephen C. Hadler, Jessica Leung, Amy E. Blain, Nancy McClung, Doug Campos-Outcalt, Rebecca L. Morgan, Sarah Mbaeyi, Jessica MacNeil, José R. Romero, H. Keipp Talbot, Grace M. Lee, Beth P. Bell, Kathleen Dooling

**Affiliations:** ^1^CDC COVID-19 Response Team; ^2^Epidemic Intelligence Service, CDC; ^3^University of Arizona, College of Medicine, Phoenix, Arizona; ^4^Department of Health Research Methods, Evidence, and Impact, Hamilton, Ontario, Canada; ^5^Arkansas Department of Health; ^6^Vanderbilt University School of Medicine, Nashville, Tennessee; ^7^Stanford University School of Medicine, Stanford, California; ^8^University of Washington, Seattle, Washington.

On February 27, 2021, the Food and Drug Administration (FDA) issued an Emergency Use Authorization (EUA) for the Janssen COVID-19 (Ad.26.COV2.S) vaccine (Janssen Biotech, Inc, a Janssen Pharmaceutical company, Johnson & Johnson; New Brunswick, New Jersey). The Janssen COVID-19 vaccine is a recombinant, replication-incompetent adenovirus serotype 26 (Ad26) vector vaccine, encoding the stabilized prefusion spike glycoprotein of SARS-CoV-2, the virus that causes COVID-19 (*1*). Vaccination with the Janssen COVID-19 vaccine consists of a single dose (5 × 10^10^ virus particles per 0.5-mL dose) administered intramuscularly. On February 28, 2021, the Advisory Committee on Immunization Practices (ACIP) issued an interim recommendation* for use of the Janssen COVID-19 vaccine in persons aged ≥18 years for the prevention of COVID-19. This vaccine is the third COVID-19 vaccine authorized under an EUA for the prevention of COVID-19 in the United States (*2*). To guide its deliberations regarding the vaccine, ACIP used the Evidence to Recommendations (EtR) framework,^†^ following the Grading of Recommendations Assessment, Development and Evaluation (GRADE) approach.^§^ The ACIP recommendation for the use of the Janssen COVID-19 vaccine under an EUA is interim and will be updated as additional information becomes available.

Since June 2020, ACIP has convened 11 public meetings to review data on the epidemiology of COVID-19 and the potential use of COVID-19 vaccines, including the Janssen COVID-19 vaccine (*3*). The COVID-19 Vaccines Work Group, comprising experts in infectious diseases, vaccinology, vaccine safety, public health, and ethics, has held weekly meetings to review COVID-19 surveillance data, evidence for vaccine efficacy and safety, and implementation considerations for COVID-19 vaccines. Within the EtR framework for the Janssen COVID-19 vaccine, ACIP considered the importance of COVID-19 as a public health problem, as well as resource use, benefits and harms, patients’ values and preferences, acceptability, feasibility, and equity. After a systematic review of available data, the work group used the GRADE approach to assess the certainty of evidence for outcomes related to the vaccine, rated on a scale of 1 (high certainty) to 4 (very low certainty) (*4*). Work group conclusions regarding certainty of evidence for the Janssen COVID-19 vaccine were discussed at public ACIP meetings (*3*).

The body of evidence for the Janssen COVID-19 vaccine was primarily informed by one international Phase III clinical trial initiated in September 2020 that enrolled approximately 40,000 participants aged 18–100 years (median age = 52 years), using two coprimary endpoints: prevention of symptomatic, laboratory-confirmed^¶^ COVID-19 among persons without evidence of previous SARS-CoV-2 infection** occurring 1) ≥14 days and 2) ≥28 days after vaccination (*5*). Interim findings from this clinical trial indicate that the Janssen COVID-19 vaccine efficacy against symptomatic, laboratory-confirmed COVID-19 was 66.3% (95% confidence interval [CI] = 59.9%–71.8%) ≥14 days after vaccination and 65.5% (95% CI = 57.2%–72.4%) ≥28 days after vaccination. At ≥14 days after vaccination, efficacy of ≥63.0% was observed across age, sex, race,^††^ and ethnicity categories and among persons with underlying medical conditions. Efficacy varied geographically and was highest in the United States (74.4%; 95% CI = 65.0%–81.6%), followed by Latin America (64.7%; 95% CI = 54.1%–73.0%) and South Africa (52.0%; 95% CI = 30.3%–67.4%). Regional differences in SARS-CoV-2 variants were noted; in South Africa, 94.5% of virus sequences from trial participants were from the B.1.351 lineage, whereas in Brazil, the P.2 lineage accounted for 69.4% of virus sequences. Vaccine efficacy for the prevention of COVID-19–associated hospitalization was high: overall, 31 COVID-19–associated hospitalizations were documented ≥14 days after vaccination, including 29 in the placebo group and two in the vaccine group (estimated efficacy = 93.1%; 95% CI = 71.1%–98.4%). No COVID-19–associated hospitalizations occurred ≥28 days after vaccination in the vaccine group, and 16 occurred in the placebo group (vaccine efficacy = 100%; 95% CI = 74.3%–100.0%). Vaccine efficacy against all-cause death was 75.0% (95% CI = 33.4%–90.6%). Seven COVID-19–associated deaths occurred, all in placebo recipients. Preliminary data suggest that the Janssen COVID-19 vaccine might also provide protection against asymptomatic SARS-CoV-2 infection,^§§^ as measured by seroconversion to a non–spike protein. Among a subset of participants with SARS-CoV-2 serology results 71 days after vaccination, 0.7% of vaccine recipients had no symptoms of COVID-19 but had documented seroconversion to a non–spike protein, compared with 2.8% of placebo recipients (estimated efficacy = 74.2%; 95% CI = 47.1%–88.6%). 

Vaccine recipients frequently experienced reactogenicity symptoms, defined as solicited local injection site or systemic adverse reactions during the 7 days after vaccination; however, the symptoms were mostly mild to moderate and resolved 1–2 days after vaccination. Symptoms were more frequent among persons aged 18–59 years than among those aged ≥60 years. Severe local or systemic reactogenicity symptoms (grade ≥3)^¶¶^ were more common in vaccine recipients than in placebo recipients (2.2% versus 0.7%). The frequency of reported serious adverse events*** was low (0.4%) both in vaccine and placebo recipients. Three serious adverse events were determined by FDA to be related to vaccination (injection site pain, hypersensitivity, and systemic reactogenicity). No specific safety concerns were identified in subgroup analyses by age, race, ethnicity, underlying medical conditions, or previous SARS-CoV-2 infection. A detailed summary of safety data, including information on reactogenicity, is available at https://www.cdc.gov/vaccines/covid-19/info-by-product/janssen/reactogenicity.html.

From the GRADE evidence assessment, the level of certainty for the benefits of the Janssen COVID-19 vaccine was type 2 (moderate certainty) for the prevention of symptomatic COVID-19. Evidence was also type 2 (moderate certainty) for the estimate of prevention of COVID-19–associated hospitalization and death. Evidence was type 3 (low certainty) for the estimates of prevention of SARS-CoV-2 seroconversion. Regarding certainty of evidence for possible harms after vaccination, evidence was type 1 (high certainty) for reactogenicity and type 2 (moderate certainty) for serious adverse events. Data reviewed within the EtR framework supported the use of the Janssen COVID-19 vaccine. ACIP determined that COVID-19 is a major public health problem and that use of the Janssen COVID-19 vaccine is a reasonable and efficient allocation of resources. Although there was variability in how populations value receipt of a COVID-19 vaccine, it was determined that for most populations, the desirable effects outweigh the undesirable effects, making the Janssen COVID-19 vaccine acceptable to implementation stakeholders. The Janssen COVID-19 vaccine is feasible to implement, requiring only a single dose and refrigerator temperatures (36°F–46°F [2°C–8°C]) for transportation and storage. These characteristics will allow for expanded availability of the Janssen COVID-19 vaccine in most community settings and mobile sites when this vaccine becomes more widely available. In addition, persons who want to complete their vaccination schedule quickly or who might have difficulty returning for a second dose might prefer a single-dose vaccine. The feasibility of administering the Janssen COVID-19 vaccine in a wider variety of settings provides an opportunity to improve equitable access to an effective COVID-19 vaccine. However, advancing health equity, particularly in populations who experience disproportionate COVID-19 morbidity and mortality, requires engagement with community leaders to identify and remove barriers to COVID-19 vaccination, including those related to vaccine access and vaccine confidence. Community engagement and education will be important as new COVID-19 vaccines are authorized for use. The GRADE evidence profile and supporting evidence for the EtR framework are available at https://www.cdc.gov/vaccines/acip/recs/grade/covid-19-janssen-vaccine.html and https://www.cdc.gov/vaccines/acip/recs/grade/covid-19-janssen-etr.html.

The Janssen COVID-19 vaccine is not interchangeable with other COVID-19 vaccine products. ACIP does not state a product preference; persons may receive any ACIP-recommended COVID-19 vaccine and are encouraged to receive the earliest vaccine available to them. Before vaccination, the EUA Fact Sheet should be provided to recipients and caregivers. Providers should counsel Janssen COVID-19 vaccine recipients about expected systemic and local reactogenicity. Additional clinical considerations are available at https://www.cdc.gov/vaccines/covid-19/info-by-product/clinical-considerations.html. Considerations for implementation are available at https://www.cdc.gov/vaccines/covid-19/phased-implementation.html. The interim recommendation and clinical considerations are based on use of the Janssen COVID-19 vaccine under an EUA and might change as more evidence becomes available. ACIP will continue to review additional data as they become available; updates to recommendations or clinical considerations will be posted on the ACIP website (https://www.cdc.gov/vaccines/hcp/acip-recs/vacc-specific/covid-19.html). 

## Reporting of Vaccine Adverse Events

FDA requires that vaccination providers report vaccination administration errors, serious adverse events, cases of multisystem inflammatory syndrome, and cases of COVID-19 that result in hospitalization or death after administration of COVID-19 vaccine under an EUA (*6*). Adverse events that occur after receipt of any COVID-19 vaccine should be reported to the Vaccine Adverse Events Reporting System (VAERS). Information on how to submit a report to VAERS is available at https://vaers.hhs.gov/index.html or 1-800-822-7967. Any person who administers or receives a COVID-19 vaccine is encouraged to report any clinically significant adverse event, whether or not it is clear that a vaccine caused the adverse event. In addition, CDC has developed a new, voluntary smartphone-based online tool (v-safe) that uses text messaging and online surveys to provide near real-time health check-ins after receipt of a COVID-19 vaccine. CDC’s v-safe call center follows up on reports to v-safe that include possible medically significant health events to collect additional information for completion of a VAERS report. Information on v-safe is available at https://www.cdc.gov/vsafe.

**Summary**

**What is already known about this topic?**

On February 27, 2021, the Food and Drug Administration issued an Emergency Use Authorization (EUA) for the Janssen COVID-19 vaccine.

**What is added by this report?**

On February 28, 2021, after a transparent evidence-based review of all available data, the Advisory Committee on Immunization Practices (ACIP) issued an interim recommendation for use of the Janssen COVID-19 vaccine in persons aged ≥18 years for the prevention of COVID-19.

**What are the implications for public health practice?**

The Janssen COVID-19 vaccine has high efficacy against COVID-19–associated hospitalization and death. Persons may receive any ACIP-recommended COVID-19 vaccine and are encouraged to receive the earliest vaccine available to them. Use of all EUA-authorized COVID-19 vaccines is critical in controlling the pandemic.

## Footnotes

* On February 28, 2021, ACIP voted 12–0 in favor of the interim recommendation for use of the Janssen COVID-19 vaccine. One ACIP member recused himself from voting because of recent (<6 months) participation in clinical trials or other studies involving companies producing COVID-19 vaccines.

†
https://www.cdc.gov/vaccines/acip/recs/grade/downloads/ACIP-evidence-rec-frame-508.pdf

§ https://www.cdc.gov/vaccines/acip/recs/grade/about-grade.html

¶ Symptomatic, laboratory-confirmed moderate to severe/critical COVID-19, defined as 1) a positive polymerase chain reaction (PCR) test result and 2) one or more of the following: respiratory rate ≥20 breaths/min, abnormal oxygen saturation, pneumonia, deep vein thrombosis, and shortness of breath or difficulty breathing or two or more of the following: fever (≥100.4°F [38°C]), heart rate ≥90 beats/min, shaking chills, sore throat, cough, malaise, headache, myalgia, gastrointestinal symptoms (e.g., nausea, vomiting, diarrhea, or abdominal pain), olfactory or taste disorder, or red or bruised toes. This definition captured almost all cases of symptomatic COVID-19 given the very limited number of mild COVID-19 cases. Laboratory confirmation of COVID-19 cases with PCR testing could have been performed at local laboratories, the central laboratory at University of Washington, Covance, or laboratories external to the study. All PCR assays were authorized by FDA. The sponsor’s two coprimary endpoints were vaccine efficacy of moderate to severe/critical COVID-19 only confirmed by the central laboratory 1) ≥14 days and 2) ≥28 days after vaccination. This GRADE review conducted by CDC was based on vaccine efficacy of any PCR-confirmed moderate to severe/critical COVID-19 case ≥14 days after vaccination. The vaccine efficacy estimated using all PCR-positive cases was not meaningfully different from the efficacy using protocol-specified PCR confirmation by a central laboratory, which was not yet available for all cases at the time of analysis.

** Persons with positive serology test results at baseline were excluded from the primary efficacy analyses but were included in all safety analyses. Efficacy was similar in a secondary analysis that included participants both with and without evidence of previous SARS-CoV-2 infection.

†† Defined as White or Black race; numbers for other race groups were too small to produce reliable estimates.

§§ Asymptomatic SARS-CoV-2 infection is defined as 1) a positive antibody test (to a non–spike protein), and 2) no previous positive SARS-CoV-2 PCR test result or COVID-19 symptoms during the study. Seroconversion to a non–spike protein can be used to distinguish between natural infection and vaccine-induced immunity.

¶¶ Grade 3 reactions are defined as those requiring use of a prescription pain reliever or preventing daily activity or a fever of 102.1°F–104.0°F (39°C–40°C); grade 4 reactions are defined as those requiring hospitalization or preventing basic self-care or fever >104.0°F (40°C). No grade 4 reactions were reported.

*** Serious adverse events are defined as any untoward medical occurrence that results in death, is life-threatening, requires inpatient hospitalization or prolongation of existing hospitalization, or results in persistent disability or incapacity; suspected transmission of any infectious agent via a medicinal product; and a medically important event.

## References

[1] Food and Drug Administration. Janssen COVID-19 vaccine emergency use authorization. Silver Spring, MD: US Department of Health and Human Services, Food and Drug Administration; 2021. https://www.fda.gov/emergency-preparedness-and-response/coronavirus-disease-2019-covid-19/Janssen-covid-19-vaccine

[2] Food and Drug Administration. COVID-19 vaccines. Silver Spring, MD: US Department of Health and Human Services, Food and Drug Administration; 2021. https://www.fda.gov/emergency-preparedness-and-response/coronavirus-disease-2019-covid-19/covid-19-vaccines

[3] Advisory Committee on Immunization Practices. ACIP meeting information. Atlanta, GA: US Department of Health and Human Services, CDC; 2021. https://www.cdc.gov/vaccines/acip/meetings/index.html

[4] Advisory Committee on Immunization Practices. Advisory Committee on Immunization Practices (ACIP): GRADE (grading of recommendations, assessment, development and evaluation). Atlanta, GA: US Department of Health and Human Services, CDC; 2020. https://www.cdc.gov/vaccines/acip/recs/grade

[5] Vaccines and Related Biological Products Advisory Committee. Vaccines and Related Biological Products Advisory Committee February 26, 2021, meeting: FDA briefing document. Silver Spring, MD: US Department of Health and Human Services, Food and Drug Administration; 2021. https://www.fda.gov/media/146217/download

[6] Food and Drug Administration. Fact sheet for healthcare providers administering vaccine (vaccination providers). Emergency use authorization (EUA) of the Janssen COVID-19 vaccine to prevent coronavirus disease 2019 (COVID-19). Silver Spring, MD: US Department of Health and Human Services, Food and Drug Administration; 2021. https://www.fda.gov/media/146304/download

